# Neurodynamic Evidence Supports a Forced-Excursion Model of Decision-Making under Speed/Accuracy Instructions

**DOI:** 10.1523/ENEURO.0159-18.2018

**Published:** 2018-06-26

**Authors:** Laure Spieser, Carmen Kohl, Bettina Forster, Sven Bestmann, Kielan Yarrow

**Affiliations:** 1 Department of Psychology, Cognitive Neuroscience Research Unit, City, University of London, London EC1V 0HB, United Kingdom; 2 Sobell Department of Motor Neuroscience and Movement Disorders, UCL Institute of Neurology, University College London, London WC1N 3BG, United Kingdom

**Keywords:** centroparietal positivity, decision-making, motor-evoked potentials, race model, sequential sampling model, speed-accuracy tradeoff

## Abstract

Evolutionary pressures suggest that choices should be optimized to maximize rewards, by appropriately trading speed for accuracy. This speed-accuracy tradeoff (SAT) is commonly explained by variation in just the baseline-to-boundary distance, i.e., the excursion, of accumulation-to-bound models of perceptual decision-making. However, neural evidence is not consistent with this explanation. A compelling account of speeded choice should explain both overt behavior and the full range of associated brain signatures. Here, we reconcile seemingly contradictory behavioral and neural findings. In two variants of the same experiment, we triangulated upon the neural underpinnings of the SAT in the human brain using both EEG and transcranial magnetic stimulation (TMS). We found that distinct neural signals, namely the event-related potential (ERP) centroparietal positivity (CPP) and a smoothed motor-evoked potential (MEP) signal, which have both previously been shown to relate to decision-related accumulation, revealed qualitatively similar average neurodynamic profiles with only subtle differences between SAT conditions. These signals were then modelled from behavior by either incorporating traditional boundary variation or utilizing a forced excursion. These model variants are mathematically equivalent, in terms of their behavioral predictions, hence providing identical fits to correct and erroneous reaction time distributions. However, the forced-excursion version instantiates SAT via a more global change in parameters and implied neural activity, a process conceptually akin to, but mathematically distinct from, urgency. This variant better captured both ERP and MEP neural profiles, suggesting that the SAT may be implemented via neural gain modulation, and reconciling standard modelling approaches with human neural data.

## Significance Statement

Successful organisms need to make the right choice fast. To make such decisions, we are regularly forced to trade speed for accuracy. This tradeoff has been explained in behavioral models using a single free parameter reflecting response caution. However, neural evidence suggests that more widespread changes are associated with quick versus accurate decisions. Here, we suggest a model which reconciles these seemingly contradictory findings. This “forced-excursion” model is mathematically equivalent to standard models of response caution but implies a global modulation in activity akin to a change in neural gain or urgency. Re-expressed in this way, the model is able to account for both behavioral and neural data from two separate neural recording techniques.

## Introduction

Every day, we make countless decisions, each requiring an appropriate compromise between speed and accuracy. This speed-accuracy tradeoff (SAT; Garrett, 1922; Hick, 1952; Wickelgren, 1977) appears ubiquitous across experimental tasks and species (Chittka et al., 2003; Ivanoff et al., 2008; Heitz and Schall, 2012). The process of making decisions can be formally described using sequential sampling models: sensory evidence accumulates over time, until a decision boundary is reached, triggering a response (Ratcliff, 1978; Brown and Heathcote, 2008). Such models traditionally explain SAT-related changes in the reaction-time distributions of both correct and erroneous responses by adjusting their boundary parameter. This reduces the required accumulation excursion, leading to faster but more error-prone decisions (Usher and McClelland, 2001; Smith and Ratcliff, 2004; Bogacz et al., 2006; Brown and Heathcote, 2008).

Signals displaying the accumulation predicted by these models have been identified in electrophysiological data from nonhuman primates (Shadlen and Newsome, 1996, 2001; Gold and Shadlen, 2000) and recently also in humans (Donner et al., 2009; O’Connell et al., 2012; Hadar et al., 2016). However, when instructions or payoffs change, neural accumulation profiles appear inconsistent with a changing boundary, the traditional model-based explanation of the SAT (Heitz and Schall, 2012, 2013; Hanks et al., 2014).

Hanks et al. (2014) proposed that the SAT is explained by an urgency signal in monkeys. Similarly, a recent human neuroimaging study proposed that urgency may arise from a global modulation of neural gain (Murphy et al., 2016). In fact, the concept of an evidence-independent urgency signal, which increases over time to inflate the accumulation process, has been a recurring theme in the recent SAT literature (Cisek et al., 2009; Milosavljevic et al., 2010; Thura et al., 2012). This urgency signal may increase faster under speed instructions, leading to faster, more error-prone responses. However, alternative accounts, prioritising human behavioral data, favor models which implement boundary differences (hereafter referred to as “classic” models) as opposed to urgency signals (Hawkins et al., 2015a,b; see also Evans et al., 2017).

Here, we aimed to square these contrasting behavioral and neural findings. In classic models, the use of a varying boundary to explain the SAT is in fact merely a conceptually appealing convention. Since sequential sampling models are formally nonidentifiable (i.e., different parameter combinations can yield the same prediction), one parameter must be chosen as a scaling parameter and fixed to an arbitrary value (i.e., changing its value will lead to a change in the value of all parameters but not in their relation to each other and therefore will not affect the model fits; Ratcliff and Rouder, 1998; Donkin et al., 2009a). This suggests that a variant of the classic model could be used to transfer the effects of the SAT onto other model parameters, while providing an equivalent fit to the data. We hypothesized that this mathematical sleight of hand would reconcile the classic bound-variation explanation of the SAT with neural findings.

We tested this hypothesis against data from two experiments. Experiment 1 used transcranial magnetic stimulation (TMS) to track corticospinal excitability, a downstream signal presumed to be under continuous influence from the decision variable (Bestmann et al., 2008; Duque et al., 2010; Hadar et al., 2016; Klein-Flugge and Bestmann, 2012). In experiment 2, we recorded the event-related potential (ERP) centroparietal positivity (CPP; O’Connell et al., 2012; Kelly and O’Connell, 2013; Twomey et al., 2016), a large, late positivity recorded over parietal regions. Importantly, this ERP has been suggested to reflect decision-related accumulation directly, independently of associated motor responses. These ERP and motor-evoked potential (MEP) signals therefore represent fundamentally different neural generators, which have both been shown to reflect decision-making processes. We believe that this methodological triangulation permits a more robust interpretation that spans the sensorimotor pipeline.

In both experiments, participants made decisions with two difficulty levels under SAT instructions. Difficulty influences the rate of evidence accumulation (Ratcliff and McKoon, 2008; Donkin et al., 2011) and was introduced here to confirm that our signals represented plausible correlates of the decision variable. We then constructed accumulation profiles predicted when the SAT is modeled through boundary variations, and by our alternative forced-excursion approach. By comparing these neurodynamic predictions to data, we demonstrate that classic models re-expressed to have a fixed excursion provide compelling approximations to both brain and behavioral measures in humans.

## Materials and Methods

### Participants

For the TMS experiment, an opportunity sample of 22 participants (13 female), primarily students and staff at City, University of London were recruited. According to criteria established before the experiment, participants were excluded if they were unable to reach a calibrated coherence level of <90% for either of the difficulty conditions (see below, Difficulty calibration). The remaining 18 participants (11 female, mean age of 29.82, SD = 8.38) took part in three sessions, each lasting between 2 and 2.5 h and involving the same conditions (speed/accuracy easy/hard, see below). For the EEG experiment, we recruited 26 participants (17 females). Of these, 23 (15 females), with a mean age of 29.39 (SD = 7.47), pretested sufficiently well to proceed to the main experiment, and thus participated in a single 2-h session. All participants were paid £8 per hour and an additional reward for task performance (up to £4 per session). The experiments were approved by the City, University of London Psychology Department Ethics Committee.

### Stimuli and procedure

#### Stimuli and experimental setup

**Figure 1.**
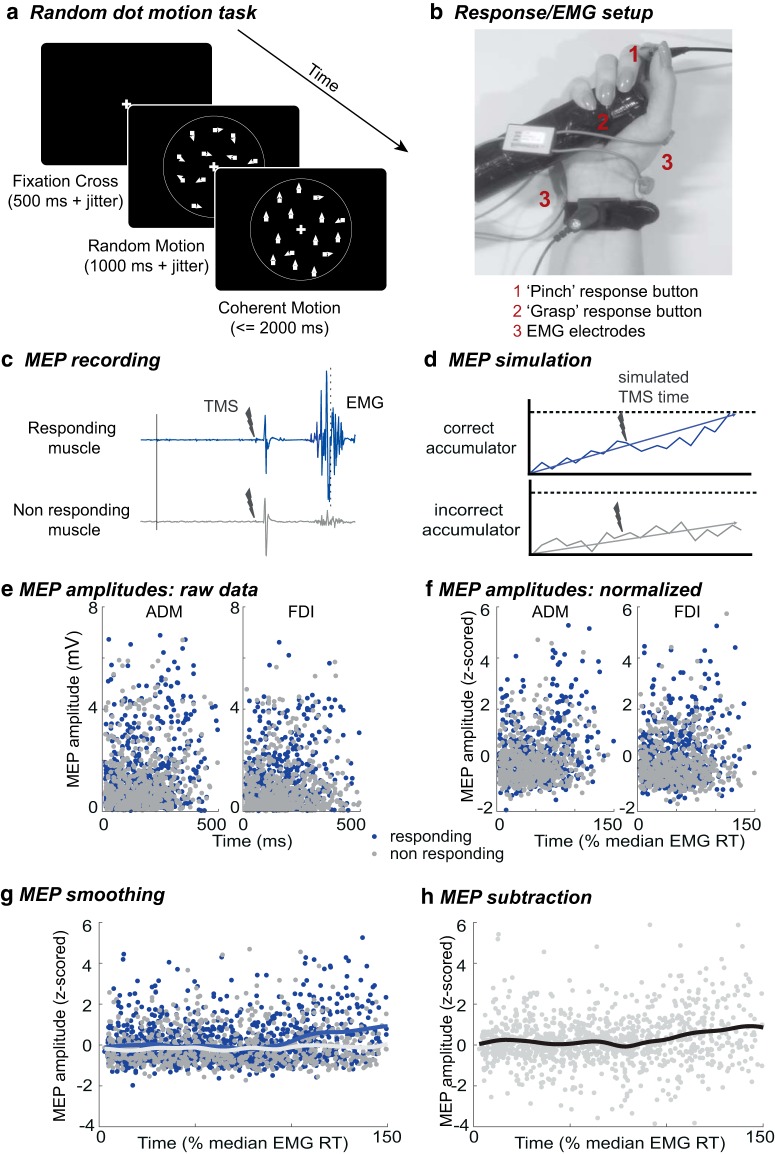
TMS experiment procedure. ***A***, Random dot motion task: after a fixation cross and a period of random motion, coherent motion (here: upward, coherence 70%) was displayed for 2000 ms or until response (the same task was used in the EEG experiment). ***B***, Response setup in TMS experiment: participants held one button (up) between their thumb and index finger (pinch) and one in the palm of their hand (down), attached to a cylinder (grasp); EMG electrodes were placed on the ADM and FDI. ***C***, Example EMG traces from a single trial (here, a hard speed trial, where the responding muscle is the FDI and the nonresponding muscle is the ADM). ***D***, To create model predictions which are comparable to MEP data, accumulation values from both the correct accumulator (corresponding to the responding muscle) and the incorrect accumulator (corresponding to the nonresponding muscle) are sampled at simulated TMS times. ***E***, Illustrative real MEP amplitudes (from the speed/easy condition) collated from all participants. ***F***, MEPs and simulations (data not shown) are then z-scored per muscle, participant, and session (note that latencies were normalized by the median, not maximum, EMG RT for each participant). ***G***, Real and simulated continuous signals can be created for each muscle (responding, nonresponding), using a Gaussian smoothing kernel. ***H***, However, to remove nonspecific processes, the same smoothing is applied to the difference between simultaneously recorded MEPs (responding minus nonresponding).

In the random dot motion task (Fig. 1*A*), participants saw an array of moving dots, a proportion of which moved coherently in one direction (equiprobably up or down) while the rest moved in random directions (selected for each dot on each frame). Trial difficulty was manipulated by varying the proportion of dots moving coherently. The task was displayed on a cathode ray tube (CRT) screen (size: 41 × 30 cm), operating at a refresh rate of 85 Hz and a resolution of 1240 × 786 pixels. Participants sat at a distance of 100 cm from the screen. In each trial, 300 white dots, each 0.04 × 0.04 degrees visual angle (dva) in size, were displayed within a 5-dva aperture on a black background. A fixation cross (size: 0.33 × 0.33 dva) was located centrally. All dots moved at a speed of 3.3 dva/s. The position of all dots was randomized every five frames. The experiment was coded in MATLAB (MathWorks), using the psychophysics toolbox extension (Brainard, 1997; Pelli, 1997; Kleiner et al., 2007) and run on a PC.

Initially, participants saw a fixation cross for 500 ms (plus a jitter of up to 1000 ms, drawn from a uniform distribution). Then, 100% of the dots moved randomly for 1000 ms (plus a jitter of up to 1500 ms, drawn from a truncated gamma distribution with shape parameter 1 and scaling parameter 150). This was followed by the onset of coherent motion, either upwards or downwards, for up to 2000 ms, or until response. Feedback was provided after each trial (see below, SAT instructions). Two equiprobable coherence levels generated “easy” (high coherence) and “hard” (low coherence) trials, which were randomly intermixed. The “speed” and “accuracy” conditions were blocked. The order of these SAT blocks was counterbalanced across participants.

Each participant completed a minimum of 100 practice trials, followed by 200 calibration trials (see below, Difficulty calibration). In each experimental TMS (EEG) session, a total of 432 (800) planned trials were completed, and self-timed breaks were provided after every 50 (100) trials. In TMS sessions, to ensure the required frequency of pulses (<0.2 Hz), TMS-free trials were added when necessary (see below, TMS and EMG processing), leading to an average of ∼500 trials per session.

#### Responses

Participants in the TMS experiment held two digital response buttons interfaced via a 16-bit A/D card (National Instruments X-series PCIe-6323, sample rate 100,000 Hz) in their right hand. One button was placed between the thumb and index finger and required a “pinch” response, contracting the first dorsal interosseous (FDI) muscle. The second button was placed on a plastic cylinder in the palm of the hand and required a “grasp” response, contracting the abductor digiti minimi (ADM) muscle (Fig. 1*B*). The pinch and grasp buttons indicated “up” and “down” responses, respectively. In the EEG experiment, participants held one button between the thumb and index finger of each hand, with right- and left-hand button presses indicating upward and downward motion, respectively.

#### Difficulty calibration

Once participants felt comfortable with the task, they completed a total of 200 staircase trials to calibrate the level of difficulty appropriate for the easy and hard conditions. A QUEST procedure (Watson and Pelli, 1983) estimated the coherence levels at which each participant responded correctly in 75% and 95% of trials, used for the hard and easy conditions, respectively. The stimulus presentation time was reduced from 2000 to 1300 ms, and no feedback was provided during QUEST trials. If a participant’s performance led to estimated hard coherence levels of >90%, the participant was excluded from the experiment. This procedure resulted in a mean coherence of 23.81% in the hard condition and 65.41% in easy trials in the TMS experiment, and 30.63% for hard, and 67.67% for easy trials in the EEG experiment.

#### SAT instructions

After the difficulty calibration, the main experiment began, in which, participants were instructed to react either as fast or accurately as possible in different blocks. Additionally, feedback was provided after each trial to either reward participants (by display of the word “correct” and a small monetary reward, adding up to a maximum of £4 per participant) for fast and correct/correct responses in speed/accuracy trials, respectively, or provide negative feedback (with the words “too slow” or “incorrect” in green letters on a red screen) when the instructions were not followed. The intertrial interval was increased by 1000 ms after each trial with negative feedback. Neutral feedback (no monetary reward but a neutral screen with the words “incorrect” or “too slow”) was shown when participants responded fast but incorrectly in the speed condition or accurately but very slowly in the accuracy condition. Whether a response was too slow or not was determined by a variable deadline, which was initially set to 600 ms for the speed and 1000 ms for the accuracy condition. To optimize performance, the deadlines varied between 450 and 750 ms (speed) and between 700 and 1300 ms (accuracy) and were adjusted using separate QUEST procedures, targeting accuracy levels of 75% for speed, and 90% for accuracy conditions. Feedback was also provided when participants responded before the onset of the coherent motion (“too fast”).

#### TMS and EMG processing

In the TMS experiment, participants’ muscle activity was recorded using surface electromyography (EMG), sampled at 1000 Hz via a 13-bit A/D Biometrics Datalink system (version 7.5, Biometrics Ltd.). We placed 22 × 28-mm surface Ag/AgCL electrodes on the skin above the FDI and the ADM of the right hand, as they contribute to the pinch and grasp responses, respectively. Reference electrodes were placed at distances of ∼2 cm to each active electrode. Participants were instructed to relax their hand muscles in between responses, and the EMG signals were passed to two speakers to provide auditory feedback about any unwanted muscle activation.

During the experiment, single-pulse TMS was applied using a Magstim Rapid^2^ biphasic stimulator (Magstim Co Ltd.). A figure-of-eight coil was positioned over the optimal spot on the scalp over the left primary motor cortex to elicit MEPs in both the ADM and FDI. The exact location was adjusted for each participant and the stimulation intensity was set at ∼110% of the resting motor threshold, to evoke potentials of around 1 mV in both muscles. The resting motor threshold was defined as the minimal intensity necessary to elicit a MEP with a peak-to-peak amplitude of ∼50 μV in 50% of stimulations in both the FDI and the ADM, and was, on average, 59.28% (SD = 7.76) of maximum stimulator output.

TMS pulses were planned in 66% of trials but cancelled if a response was detected before stimulation. To ensure a good distribution of TMS pulses over the course of the reaction time, TMS trials were divided into four equally sized, equiprobable time bins (between 5 and 500 ms relative to the onset of the coherent motion in the speed condition and between 5 and 600 ms in the accuracy condition). Within a given bin, the exact stimulation time was drawn uniform randomly. Since the experiment followed a single-pulse TMS protocol, pulses were required to occur at a maximal frequency of 0.2 Hz. If, by chance, a planned pulse followed a previous one after <5000 ms, the task was adjusted in several ways. If the timespan between the previous and the planned pulse was <5000 ms but >4000 ms, the intertrial interval was increased to decrease the pulse frequency to <0.2 Hz. For scheduled intervals of <4000 ms, the planned trial was replaced with the next planned stimulation-free trial. If no stimulation-free trial remained, random stimulation-free trials were generated to increase the interval between TMS pulses, resulting in an average of 68.67 (SD = 1*5*.79) additional trials per session.

#### EMG preprocessing

To eliminate potential differences in the time required to execute pinch and grasp responses, we recorded the onset of EMG as a measure of reaction time (EMG RT). EMG data from both channels were aligned to the onset of the coherent motion (stimulus onset) and visually inspected to select the onset of response-related EMG bursts. Visual inspection provided no information about the experimental condition of a given trial.

In TMS trials, MEP amplitudes in both channels (FDI and ADM) of the right hand were defined as the difference between the minimal and maximal EMG values in a time window of 10–40 ms relative to stimulation time. An algorithm detected EMG activity before the stimulation, discarding any trials in which there was activity >50 μV peak to peak in a period of 200 ms preceding the stimulation. These trials, as well as trials in which there was partial activation in more than one channel, or trials in which a clear EMG onset could not be detected, were excluded from further analysis (23.39% of trials). Additionally, trials with very fast (<100 ms) or very slow (>1800 ms) response onsets (5.12% of trials), trials in which no MEP was visible or in which the MEP amplitude could not be accurately detected due to amplifier saturation (1.05%), and trials in which the response preceded the planned TMS pulse (6.09%) were excluded. In total, 35.65% of all trials were discarded, with a total of 17,067 trials remaining, including 6535 usable TMS trials (42.85% of all planned TMS trials).

#### MEP processing

To yield sufficient data to accurately estimate corticospinal excitability in a time-continuous manner, correct-trial MEPs from all participants were combined. Before pooling, MEP amplitudes were z-transformed separately for each muscle, session and participant, while TMS latencies were normalized by median RT of TMS-free trials in the corresponding session. Z-scored MEPs were then sorted as a function of stimulation latency (Fig. 1*C*,*E*,*F*) and smoothed using a Gaussian kernel to recover a continuous time-varying MEP average in steps of 1% median RT:
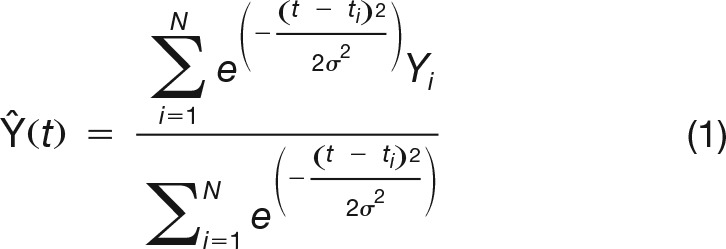


Where the *N* contributing MEPs each have amplitude *Y_i_* and occur at normalized time *t_i_*. The width of the Gaussian kernel defined by the full width half maximum was set at 5% of median RT (i.e., around 20 ms), previously suggested as an appropriate compromise between temporal resolution and signal-to-noise ratio (Hadar et al., 2016). This MEP signal was computed for both stimulus and response-locked MEP latencies, and from the responding muscle, the nonresponding muscle and the MEP amplitude difference between them (Fig. 1*G*,*H*). Finally, 95% confidence intervals were estimated around each signal using a bias-corrected and accelerated bootstrap (BCa) confidence interval, based on 1999 iterations. Since analyses were restricted to correct trials, MEPs recorded from the responding muscle always reflected activation of the correct response, while MEPs form the nonresponding muscle reflected the incorrect response. We focused particularly on the MEP average signal based on the amplitude difference between responding and nonresponding MEPs, as this eliminates variations due to nonspecific influences, such as inhibitory processes during action preparation, which would result in MEP suppression in both responding and nonresponding muscles (for review, see Duque et al., 2017).

### EEG recording and processing

Continuous EEG was recorded using 64 active electrodes, placed equidistantly on the scalp (EasyCap, M10 Montage) and referenced to the right mastoid (BrainAmp amplifier; BrainProducts; sampling rate: 1000 Hz). The data were preprocessed and analyzed using custom scripts in MATLAB (MathWorks), drawing on functions from the EEGLAB toolbox (Delorme and Makeig, 2004).

EEG data were re-referenced to the average reference and digitally bandpass filtered (0.1–45 Hz). Data were visually inspected to remove large muscle artefacts before applying ICA to remove eye blink components. Any remaining artefacts were removed manually during a second visual inspection. Afterward, spherical spline interpolation was used to reconstruct noisy channels, which were identified and rejected during the first visual inspection. In line with the procedures used in previous CPP studies (O’Connell et al., 2012; Kelly and O’Connell, 2013), the data were converted to current source density (CSD) estimates using the CSD toolbox (Kayser and Tenke, 2006).

### Experimental design and statistical analysis

#### Behavioral data analysis

We explored the within-subjects factors instruction and difficulty with the levels speed/accuracy and easy/hard, respectively. To test their effects on RT, we used a 2 × 2 repeated-measures ANOVA. Because accuracy data violate the assumptions of ANOVA, statistical inferences about errors were made using a generalized linear mixed-effects model with a logistic link function and binomial data model (applied using the fitglme function in MATLAB). Parameter estimates were based on a maximum-likelihood method using Laplace approximation and the “maximal” random effects structure (Barr et al., 2014), i.e., both instruction and difficulty, and the instruction-difficulty interaction were entered as fixed effects, and both manipulations, and their interaction within each participant (and session in the TMS experiment) were included as random effects.

#### MEP analysis

Two analyses were conducted on the MEP difference signal to confirm that MEP modulations across time reflected decision-related accumulation processes. We compared the stimulus-locked build-up rate, expected to be steeper in easy than hard trials, and the response-locked signal amplitude, which should not vary across difficulty levels at the time of decision. Comparisons were also made across speed instructions, although no clear predictions could be made regarding how evidence accumulation should vary in this case. MEP data were permuted across easy and hard (or across speed and accuracy) trials 1999 times. Mean MEP signals (and 90% BCa confidence intervals; see below) were then computed for each iteration. The build-up rate was then estimated from both the original and the resampled data as the slope of a straight line fitted to the stimulus-locked signal in a time window ranging from half median up to median RT (corresponding to around 200–400 ms after stimulus onset). Slope differences between difficulty levels or instructions were considered significant if smaller (or larger) than the lower (or upper) 2.5% of the corresponding slope-difference null distribution obtained from resampled signals.

To test response-locked amplitude differences while controlling for multiple comparisons, a cluster statistic was calculated (cf. Blair and Karniski, 1993; Nichols and Holmes, 2001; Groppe et al., 2011). Potential regions of difference between conditions were based on contiguous time periods with no overlap between 90% bootstrap BCa confidence intervals (the arbitrary “cluster threshold”). A cluster sum was calculated within each such putative cluster and was considered significant when this sum of the point-by-point differences fell outside the central 95% of the corresponding distribution of the biggest cluster sum obtained from resampled signals. Amplitude differences were assessed on both stimulus and response-locked signals.

#### ERP analysis

For the ERP analysis, we extracted both stimulus (−200–2000 ms, relative to coherent motion onset) and response aligned (−1000–100 ms, relative to the button press) epochs. All epochs were baseline corrected to the average over a 200–ms period preceding motion onset. The appropriate electrode to generate the CPP wave form was chosen individually, by visually inspecting each participant’s averaged ERP topography to identify the centroparietal region of maximum amplitude (chosen electrodes: 1, 5, or 14, roughly equivalent to electrodes Cz, CPz, Pz in the 10–20 system). The activity recorded on the selected electrode was averaged for each condition (collapsed over up and down trials) and for stimulus and response-locked signals separately. In line with Kelly and O’Connell (2013), we measured the slope of the CPP for each participant, by fitting a straight line to the wave form from 200 to 350 ms in the stimulus-locked data. Additionally, we measured the peak amplitude of the response-locked ERP by averaging over the amplitude of the wave form from -50 to 50 ms relative to the response. Differences across conditions were assessed with a 2 × 2 repeated-measures ANOVA.

### Modeling

#### Free-excursion race model

According to a standard free-excursion race model (Laberge, 1962; Vickers, 1970; Bogacz et al., 2006) evidence supporting the correct and the incorrect response is integrated independently in two accumulators. The amount accumulated at each time step (d*x*) is given by:
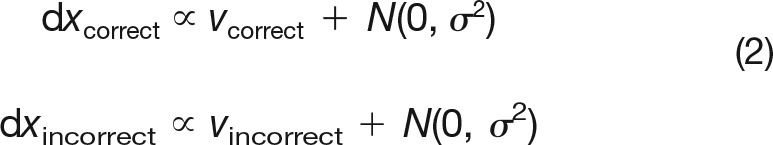


Where *x*_correct_ and *x*_incorrect_ are the quantities accumulated, and *v*_correct_ and *v*_incorrect_ the input evidence (i.e., accumulation rate; see below) in favor of the correct and the incorrect responses. Noise, *N*, drawn from a normal distribution of mean 0 and standard deviation *σ*, is also integrated at each iteration. To avoid negative values, evidence accumulated at each time step is updated as:
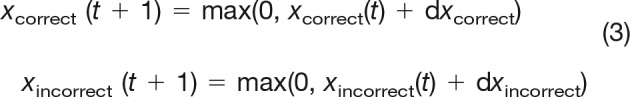


Correct and incorrect accumulator starting points are drawn in each trial from a uniform distribution ranging between 0 and *S*_Z_. As soon as one of the accumulators reaches the response boundary *A*, the corresponding response is selected. The response time is then modeled as the time required to reach the boundary, plus non-decision time, during which sensory and motor processes occur, drawn from a uniform distribution centered on *T*_er_ and of width *S*_Ter_. In a standard race model for a binary decision, this leads to a total of seven parameters (*A*, *S_z_*, *v_correct_*, *v_incorrect_*, *T_er_*, *S_Ter_*, and *σ^2^*). One parameter is chosen as a scaling parameter and fixed to an arbitrary value, resulting in a total of six free parameters.

To apply this model to the data in this experiment, we added accumulation rate parameters to account for the different difficulty conditions (*v_easy_correct_*, *v_easy_incorrect_*, *v_hard_correct_*, *v_hard_incorrect_*). This implementation of difficulty is well-established and has been validated using both behavioral and neural data (Ratcliff and Rouder, 1998; Roitman and Shadlen, 2002; Ratcliff and McKoon, 2008; Mulder et al., 2014; Twomey et al., 2015). To explain differences due to SAT instructions, we added a second boundary parameter. The boundary for accuracy trials *A_accuracy_* acted as a scaling parameter and was fixed to 1, while the boundary for the speed condition, *A_speed_*, was free to vary. We tested three different models: one in which all remaining parameters were fixed across conditions (model 1), one in which the starting point parameter *S_z_*was free to vary across SAT conditions (model 2), and one in which the non-decision time parameter *T_er_* was free to vary across SAT conditions (model 3; Table 1).

**Table 1.** Model comparison

Model Number	A	S_z_	v_correct_	v_incorrect_	T_er_	S_Ter_	σ	Number of parameters	TMS experiment		EEG experiment	
									AIC	BIC	AIC	BIC
Model 1	Free	Fixed	Fixed	Fixed	Fixed	Fixed	Fixed	9	44,868	44,933	62,398	62,466
Model 2	Free	Free	Fixed	Fixed	Fixed	Fixed	Fixed	10	**44,859**	**44,932**	**62,389**	**62,464**
Model 3	Free	Fixed	Fixed	Fixed	Free	Fixed	Fixed	10	44,865	44,937	62,404	62,479

Bayesian Information Criterion (BIC) and AIC values for each model and each experiment (best BIC and AIC values in bold). The terms “fixed” and “free” here relate specifically to changes across speed/accuracy instructions, as accumulation rate (V) was always free to vary between difficulty conditions.

Modeled RTs were simulated based on Equations 2 and 3 (10,000 simulated trials with a 1% median RT time step, around 4 ms, for TMS and a 10-ms time step for EEG) and compared to pooled RT data using quantile maximum probability estimation (Heathcote et al., 2002). Specifically, we estimated empirical RT quantiles (at 0.1, 0.3, 0.5, 0.7, and 0.9), for both correct and erroneous responses, and compared counts of simulated RTs in the resulting bins against the predicted multinomial distribution. Parameter values were adjusted using a differential evolution algorithm implemented in MATLAB (Price et al., 2005). The goodness-of-fit of the different models was assessed by computing the Akaike information criterion (AIC; Akaike, 1977).

#### Forced-excursion race model variant

To test the hypothesis that the SAT is not implemented through decision bound variation per se, but rather by more widespread changes of neural activity, we constructed a forced-excursion model variant in which decision boundaries are fixed and the effects of the SAT are transferred onto all other parameters. All parameters of the free-excursion race model estimated in the speed condition were divided by the speed boundary *A*_speed_ (apart from *T*_er_ and *S*_Ter_). This forced-excursion version of the model is mathematically equivalent to the original one as, given the scaling property of sequential sampling models, multiplying all models parameters (except *T*_er_ and *S*_Ter_) by the same amount does not affect model predictions (Donkin et al., 2009b). A simple “rescaling” of speed parameters hence results in a new set of parameters in which the speed and accuracy response boundaries are equal, and the SAT modulation is transferred onto the other decision-related parameters.

#### Model predictions

##### TMS experiment

In each session, EMG RTs were normalized by median EMG RT, and trials were pooled across sessions and participants. On average, we obtained 2651 trials per condition, used to determine best-fitting parameters at the group level. We then generated predictions according to the free and forced-excursion race model variants by simulating evidence accumulation. To allow for a direct comparison, model predictions were constructed identically to the accumulation signals derived from our experimental data, i.e., as MEP difference average signals.

For both models, and each condition, 20,000 single-trial accumulation paths were computed based on Equations 2 and 3 (in 0.5% median EMG RT time steps). Each modeled MEP amplitude was determined by the value of one of the single-trial simulated accumulation signals reached at a (simulated) TMS latency, based on stimulation times applied during the experiment (Fig. 1*D–F*). The difference between correct and incorrect values was used to model the MEP difference signal. As in experimental data, trials were discarded when simulated RT was shorter than TMS latency (i.e., the response would have been given before the TMS pulse). The duration of sensory and motor processes, which are represented by a single T_er_ parameter, had to be allocated to pre- and postaccumulation processes to generate predictions. Since we modeled accumulation observed in or around M1, we assumed that postaccumulation stages would only relate to response execution, which could reasonably be ignored, as reaction times were defined up to EMG onset. Therefore, the whole of *T*_er_ was allocated to preaccumulation processes, and accumulation started after a delay of *T*_er_ ± *S*_Ter_.

From simulated MEPs, predicted continuous MEP signals were then computed by applying the same smoothing method applied to the MEP data. Finally, accumulation signals based on predicted MEPs were compared to the empirical MEP signal using a mean squared error metric, after a scaling procedure was applied to match modeled and experimental signal amplitudes. Modeled signals were vertically normalized by the value minimizing the mean squared error, estimated using the previously described differential evolution algorithm. Note that although this normalization could differ between the free and forced-excursion models, the same value was applied within each model to all conditions, and to stimulus and response-locked signals.

Finally, two complementary statistical analyses compared the mean squared errors obtained for the free and forced-excursion model variants, to determine which predictions displayed greater similarities to the neural signal. First, goodness-of-fit of the model predictions was computed based on AIC values, using the formula AIC = n*log(MSE) + 2*K* (Burnham and Anderson, 2004), where *n* is the number of observations, MSE the mean squared error, and *K* the number of free parameters (*K* = 1 in this case, as only amplitude was allowed to vary freely to fit recorded MEP signals). AIC was then used to compute Akaike model weights, which can be seen as the weight of evidence in favor of each model.

The second analysis applied a bootstrap procedure estimating the distribution of differences of mean squared error between the free and forced-excursion models, to determine the bias-corrected 95% confidence interval around the observed difference (bias-correction was used rather than BCa to make the time of computation manageable). To estimate the distribution, EMG RT data were resampled 1999 times with replacement within each condition. The best-fitting parameters for the original and each resampled set of EMG RT data were then estimated by a simplex algorithm implemented in MATLAB (Lagarias et al., 1998), using the original parameters as starting values (the Simplex algorithm was preferred to the differential evolution algorithm in this case to reduce the time of computation). As for the original analysis, forced-excursion parameters were obtained by normalizing the free-excursion parameters by the response boundary value obtained in the speed condition, and MEP signal predictions for free and forced-excursion models were computed. Mean squared errors were then calculated between these bootstrapped signal predictions and a set of equivalently resampled MEP signals, again after applying a scaling procedure matching signals amplitudes (via a differential evolution algorithm; Price et al., 2005). The 95% bias-corrected confidence interval was estimated based on the bootstrap distribution of mean squared error differences between the free and forced-excursion models.

##### EEG experiment

RTs were pooled across participants to fit the models at a group level. As EEG signals integrate spatially disparate underlying neuronal activity, we reasoned that the CPP would likely represent the sum of evidence accumulators across time. The corresponding accumulation signals predicted by the models should therefore be obtained by adding up the correct and incorrect accumulators’ activities. For both models and each speed and coherence level condition, 10,000 single-trial accumulation paths were computed based on Equations 2 and 3. To account for sensory processes, accumulation started after a sensory delay. Once a decision was made, we assumed that evidence accumulation continued until the response was executed (and the stimulus was turned off). Accumulation therefore continued after the boundary was reached for the duration of any motor processes (Resulaj et al., 2009; Twomey et al., 2015). The compound duration of sensory and motor processes were given by the model non-decision time *T*_er_, which we divided into *T*_e_ and *T*_r_, modeling sensory and motor processes, respectively. As detailed below, this division was optimized for each model. To match with EEG processing, the sum-of-accumulations signal was baseline corrected by subtracting the first data point value from each trial. Finally, to compare the prediction to the CPP, we averaged accumulation signals in each condition, either time-locked on stimulus onset (i.e., time 0), or on response time (the time of the corresponding simulated RT). Since we can only speculate on how the accumulator behaves once the response is executed, trials were removed from averaging once the simulated response time had been reached (and the same procedure was used for the averaging of empirical EEG data).

The similarity between the CPP and the predicted decision variable of each model was quantified by computing the mean squared error between mean signals. To provide optimal CPP predictions, the amplitude of each summed signal was scaled to match the CPP amplitude, and the division of non-decision time *T*_er_ into encoding time *T*_e_ and response time *T*_r_ was determined. The optimal scaling factor and *T*_er_ division were obtained for each model signal using differential evolution (Price et al., 2005), minimizing the mean squared error.

Finally, as in the TMS experiment, a bootstrap analysis (bootstrapping both RT and EEG data) determined whether the mean squared error difference calculated for the free- and the forced-excursion models had a 95% confidence interval excluding zero, i.e., whether they differed significantly. In this experiment, no AIC-based comparison was attempted because EEG data points have complex temporal dependencies (i.e., autocorrelation) that make it difficult to establish the likelihood with which a model predicts these neurodynamic data.

## Results

### Behavioral results

**Figure 2.**
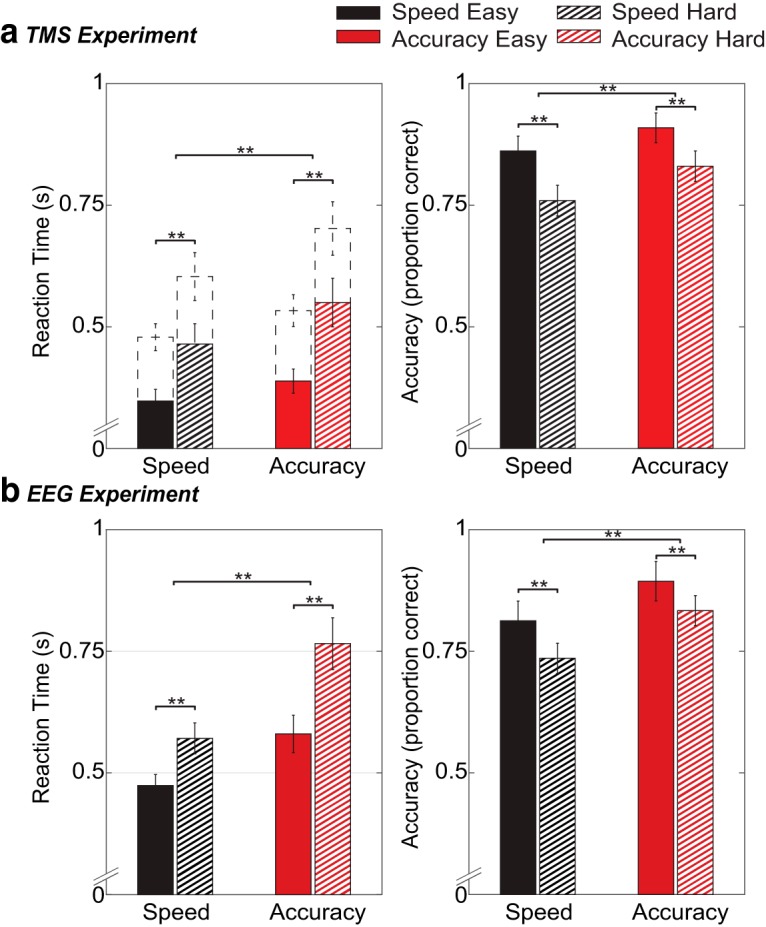
Behavioral results for both the TMS experiment (***A***) and the EEG experiment (***B***): reaction time (left) and accuracy scores (right) for each condition. Top left panel shows both EMG RT (bars) and button RT (dashed lines). Error bars indicate 95% confidence interval; **p <.001.

Trials remaining after preprocessing were collapsed over up and down trials (Fig. 2). Both experiments revealed the same behavioral effects. As expected, RTs were faster under speed than accuracy instructions (TMS: *F*_(1,17)_ = 26.90, *p* < 0.001, *η_p_^2^* = 0.61; EEG: *F*_(1,22)_ = 36.47, *p* < 0.001, *η_p_^2^* = 0.62), as well as in easy compared to hard trials (TMS: *F*_(1,17)_ = 62.14, *p* < 0.001, *η_p_^2^* = 0.79; EEG: *F*_(1,22)_ = 120.12, *p* < 0.001, *η_p_^2^* = 0.85). Additionally, instruction and difficulty interacted (TMS: *F*_(1,17)_ = 10.80, *p* = 0.004, *η_p_^2^* = 0.79; EEG: *F*_(1, 22)_ = 36.47, *p* < 0.001, *η_p_^2^* = 0.62). Follow-up *t* tests revealed that the effect of difficulty was larger in the accuracy condition (*p* < 0.001) than in the speed condition (*p* < 0.001). All reported effects in the TMS experiment are based on EMG RT (time of EMG onset), but results based on response-button RT were not qualitatively different.

For error data, a generalized linear mixed-effects model revealed higher accuracy scores under accuracy compared to speed instruction (TMS: *t*_(208)_ = 4.81, *p* < 0.001; EEG: *t*_(88)_ = 7.76, *p* < 0.001), as well as in easy trials compared to hard trials (TMS: *t*_(208)_ = 4.57, *p* < 0.001; EEG: *t*_(88)_ = 4.68, *p* < 0.001). The instruction-difficulty interaction was not significant (*p* > 0.05).

### Neural results

#### MEP-average signals

**Figure 3.**
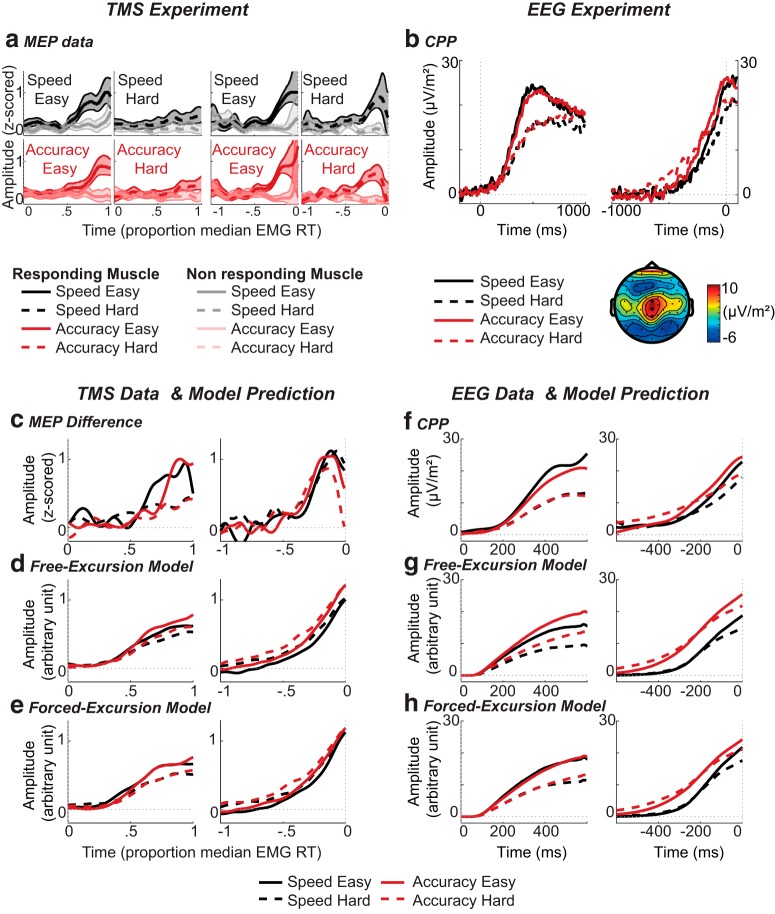
Neural and modeling results. Top, Neural data. Bottom, Model comparison. Left: TMS experiment. Right, EEG experiment. ***A***, Stimulus-locked (left) and response-locked (right) MEP signal for each condition. Each panel shows both the MEP signal associated with the responding muscle (dark) and the nonresponding muscle (light). Shaded areas indicate 95% confidence intervals. ***B***, CPP: stimulus-locked (left) and response-locked (right) CPP wave form for each condition. The bottom right of the panel shows the topography of the ERP, averaged over the stimulus-locked time interval of 0–1000 ms. Electrodes used to generate CPP waveforms are highlighted. ***C***, Stimulus-locked (left) and response-locked (right) MEP-average signal (responding minus nonresponding muscle). ***D***, Stimulus-locked (left) and response-locked (right) model predictions made by the free-excursion variant of the best-supported model. ***E***, Stimulus-locked (left) and response-locked (right) model predictions made by the forced-excursion variant of the best-supported model. ***F***, Stimulus-locked (left) and response-locked (right) CPP; note that the CPP here is a pooled average rather than a grand average and therefore differs from ***B***. Additionally, the wave form has been low-pass filtered with a cutoff of 5 Hz to assist comparison with model predictions. ***G***, Stimulus-locked (left) and response-locked (right) model predictions (correct and incorrect accumulator summed) made by the free-excursion variant of the best-supported model. ***H***, Stimulus-locked (left) and response-locked (right) model predictions (correct and incorrect accumulator summed) made by the forced-excursion variant of the best-supported model.

MEP amplitudes from correct trials were collated and smoothed to form three categories of MEP-average signal: responding, nonresponding, and the difference between them. Responding and nonresponding MEP-average signals obtained for each condition are presented in Figure 3*A*. The responding MEP-average signal (associated with the correct response) builds up gradually during the reaction-time period, while the nonresponding signal (associated with the incorrect response) remains fairly flat. However, our main focus was the difference in MEP amplitudes between responding and nonresponding muscles (Fig. 3*C*). Statistical analyses confirmed that this MEP signal displays characteristics consistent with the hypothesis that M1 excitability reflects an accumulation process. We found that the stimulus-locked signal built up faster in easy than hard trials (for both speed, *p* = 0.049, and accuracy, *p* < 0.001 instructions) and that the response-locked signal amplitude reached similar levels just before the response regardless of trial difficulty, with cluster permutation tests showing no significant divergence between conditions (*p* = 1). Differences were however observed in stimulus-locked averages, with higher amplitudes evident in easy compared to hard trials from 75% median EMG RT (∼294 ms) in the speed condition (*p* = 0.005) and from 81% (∼318 ms) under accuracy instructions (*p* < 0.001). The latter results demonstrate that we had sufficient power to detect MEP amplitude differences. Collectively, our results show that the MEP-average difference signal is a viable neural correlate of the decision variable. However, no difference was observed between speed and accuracy instructions, on either the slope or amplitude of MEP accumulation (all *p* > 0.1).

#### ERP results

The CPP is displayed in Figure 3*B*. Like the MEP-average difference signal, it builds over the course of the decision, at a rate reflecting the difficulty of the decision. For build-up rate, there was a significant main effect of difficulty (*F*_(1,22)_ = 14.70, *p* = 0.001, *η_p_^2^* = 0.40), with higher slopes in easy compared to hard trials. There was no main effect for instruction, and no interaction, in either of the time alignments (*p* > 0.26).

There was also a main effect of difficulty on the peak amplitude of the response-locked CPP, *F*_(1,22)_ = 8.53, *p* = 0.008, *η_p_^2^* = 0.28, with higher amplitudes in the easy compared to the hard conditions. However, again we found no main effect for SAT instruction and no interaction (*p* > 0.22).

Summarizing the neural data, neurodynamic signals derived from two very different imaging methods converged to yield the same outcome: clear effects of adjusting task difficulty, particularly on the rate of accumulation, but no statistically reliable effects of speed/accuracy instruction, despite the fact that these two manipulations had similar magnitudes of behavioral effect (mean RT effect sizes, i.e., *η_p_^2^,* of 0.62 for SAT instruction vs 0.82 for difficulty).

#### Model selection

**Figure 4.**
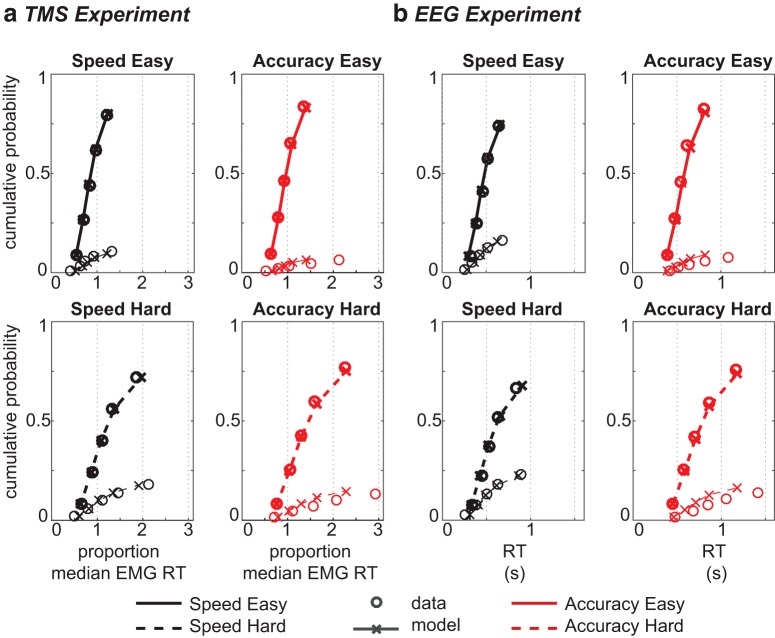
Model fit for the TMS experiment (***A***) and the EEG experiment (***B***): quantiles estimated from behavioral data (circles) and model 2 simulations (crosses and lines) for easy (top) and hard (bottom) decisions. For each condition, correct (thick) and incorrect (thin) quantiles are displayed separately. Note that the model fit is identical for the forced-excursion and the standard free-excursion race model.

In both experiments, we fitted several models to RT data and used AIC to select the best candidate with which to go on and make neural predictions. The winning race model (model 2; Table 1) varied both response boundary and starting-point between different SAT instructions (and also varied drift rates with changes in difficulty). As anticipated, the best-supported model’s best-fitting parameters (shown under “free-excursion” in Table 2) show that the response boundary decreased under speed instruction, and that accumulation rates were higher for easy than hard trials. Additionally, starting-point variability was larger under speed instructions. Since the starting-point distribution ranges from 0 to the starting-point parameter *S_z_*, larger starting-point variability also implies a larger mean starting-point, further decreasing the distance between baseline and boundary. The quality of the fit was good (Fig. 4).

**Table 2.** Estimated parameter values for the best-supported model (model 2) when expressed with both free and forced-excursion in both experiments

Parameters		TMS experiment				EEG experiment			
		Free excursion		Forced excursion		Free excursion		Forced excursion	
		Accuracy	Speed	Accuracy	Speed	Accuracy	Speed	Accuracy	Speed
*S_*Z*_*		0.447	0.523	0.447	0.586	0.319	0.541	0.319	0.664
*A*		1	0.893	1		1	0.815	1	
*T_*er*_*		0.382		0.382		0.257		0.257	
*S_*Ter*_*		0.374		0.374		0.229		0.229	
*σ^*2*^*		0.499		0.499	0.558	0.785		0.785	0.964
*v_*correct*_*	Easy	1.280		1.28	1.433	2.475		2.475	3.038
	Hard	0.634		0.634	0.710	1.350		1.350	1.656
*v_*incorrect*_*	Easy	0.098		0.098	0.109	0.253		0.253	0.310
	Hard	0.004		0.004	0.005	0.054		0.054	0.066

The response boundary A in the accuracy condition was set to 1 as a scaling parameter. Parameters are not comparable across experiments, as the TMS fit is to data normalized to the median RT of each participant.

Importantly, we also re-expressed this model under a forced-excursion constraint. In this forced-excursion version, parameter normalization forced the speed response boundary to be the same as the accuracy boundary, with the SAT being transferred onto accumulation rate and variability parameters. Note that the forced-excursion version of this model is mathematically equivalent to the standard one, with identical predicted RTs and error rates.

Stimulus and response-locked accumulation signals for each experiment and each condition predicted by the free and forced-excursion variants of the best-supported model are shown in Figure 3, lower panels. Broadly the same patterns were predicted in both experiments. The main difference between free and forced-excursion predictions is the level of accumulation reached at the time of the decision. This is evident in the amplitude of response-locked signals attained just before response selection, which is predicted to be higher under accuracy than speed instructions for the free-excursion model, but similar in the forced-excursion model (Fig. 3*D*,*E*,*G*,*H*). Note that, while this pattern is more pronounced in the forced-excursion predictions associated with the MEP signal (Fig. 3*E*) than the EEG signals (Fig. 3*H*), the reduced amplitude difference between speed and accuracy profiles before the response is evident in both experiments, and importantly, both forced-excursion model predictions capture the patterns seen in the corresponding neural data (Fig. 3*C*,*F*). In the stimulus-locked predictions, easy trials display a steeper build-up than hard trials, yet, interestingly, although accumulation rates in the forced-excursion model were higher under speed than accuracy instructions (Table 2), the predicted signal was not correspondingly steeper in this case (Fig. 3*E*,*H*). For MEPs, this may be partly explained by the fact that both correct and incorrect accumulation rates increased, such that the slope of the (motoric, thus difference-based) accumulation signal remained unaffected. However, the similar pattern observed in CPP predictions (which were modeled as a sum of accumulators, because this signal occurs relatively early and is not response specific) indicates that the ∼20% change in modeled accumulation rate was insufficient to generate a substantial increase in predicted slope when combined with the associated changes in noise parameters.

Summarizing these observations, the signals predicted by the forced-excursion version of the best-supported model appear to better reproduce the pattern of the recorded CPP and MEP signals than do those predicted by the free-excursion version. Specifically, the accumulation slope is steeper in easy than hard trials, but not different between speed and accuracy conditions, and a similar signal amplitude is attained before response for both coherence levels, and, crucially, under both SAT instructions.

Statistical analyses confirmed these observations. Akaike weights in the TMS experiment indicated that neurodynamic predictions from the forced-excursion model variant were better matched to the MEP signals than were free-excursion predictions (forced-excursion: 0.994, free-excursion: 0.006). Additionally, bootstrap analysis showed that the mean squared error between predicted MEP signals and recorded MEP values was significantly lower for the fixed than the free-excursion model (*p* = 0.018, 95% bias-corrected confidence interval on difference: [0.005; 0.056]). This significant difference, observed using a BCa confidence interval, was not however evident when a simpler percentile interval was used. This result should hence be interpreted cautiously (but is bolstered by our subsequent findings with EEG). The same bootstrap analysis revealed similar results in the EEG experiment, where the forced-excursion model predicted profiles more similar to the CPP than the free-excursion model (*p* = 0.026, 95% bias-corrected confidence interval on this difference: [1.55; 21.32]; for consistency, we repeated the model comparison for the ERP data set with RT normalized data and found that the results were unchanged).

## Discussion

We utilized two separate electrophysiological methods to explore the neurocognitive mechanisms underlying the SAT, a central yet unresolved issue in decision-making research. The model-based behavioral literature suggests that a variation in the decision boundary (or, equivalently, a change in the baseline level) explains the SAT (Usher and McClelland, 2001; Smith and Ratcliff, 2004; Brown and Heathcote, 2008), but recent neural evidence has not supported this claim, suggesting more widespread changes (Heitz and Schall, 2012, 2013; Hanks et al., 2014; Murphy et al., 2016). To resolve this paradox, we hypothesized that the SAT may result from changes which are mathematically equivalent to a modulation of the decision boundary, but which are implemented physiologically through global changes in neural activity akin to turning up the gain in the brain. We recorded neurodynamic substrates of decision-making during a motion discrimination task with two difficulty levels and under instructions to focus on either response speed or accuracy. The resulting data converged to favor the predictions made by a forced-excursion model variant in which the SAT is implemented by adjusting both the signal (i.e., accumulation rates *v*) and noise (i.e., noise parameters *S_z_* and *σ*) affecting accumulation-related neural activity.

Although our main interest was the SAT, we included a difficulty manipulation as a “sanity test” regarding the validity of our neurodynamic decision correlates. The impact of difficulty on evidence accumulation has been demonstrated previously, with both sequential sampling models and proposed neural correlates of accumulation displaying steeper build-up rates in easier decisions (Roitman and Shadlen, 2002; Ratcliff and McKoon, 2008; Kelly and O’Connell, 2013; Mulder et al., 2014). Accordingly, we found that faster and more accurate responses in easy trials were explained by higher accumulation rates in both experiments. These patterns were observed in both neural signals and their simulated accumulation profiles and, consistently with previous studies (O’Connell et al., 2012; Hadar et al., 2016), support the role of MEP and CPP signals as neural correlates of the decision variable, with corticospinal excitability likely receiving a time-lagged but continuous input from CPP/decision-generating regions.

Like the difficulty manipulation, SAT instructions also resulted in the expected behavioral changes, with faster and more error prone responses under speed instructions. In line with many previous studies (Usher and McClelland, 2001; Brown and Heathcote, 2008; Ratcliff and McKoon, 2008; Heitz, 2014), our free-excursion race model accounted for behavioral effects of the SAT, primarily by varying the amount of accumulated evidence required to make a decision. However, since recent studies exploring neural correlates of decision-making have challenged this implementation of the SAT (Heitz and Schall, 2012, 2013; Hanks et al., 2014; Murphy et al., 2016), we used a forced-excursion variant which models a global gain modulation by adjusting the parameters of the free-excursion race model so that the boundary was equal across SAT conditions, thus transferring the estimated difference between response bounds onto all other parameters affecting accumulation. In other words, a fixed boundary between SAT conditions was made mathematically equivalent to the free-excursion model by assuming different underlying mechanisms, with changes between SAT conditions explained not by boundary differences, but by differences between virtually all other parameters, modeling a global shift in decision-related brain activity.

When we compared predicted accumulation profiles from both the free and the forced-excursion model variants to our neural data, a fixed boundary provided significantly better degrees of correspondence between them (we avoid the term “goodness of fit” here, because predictions were based on RT data, with little adjustment required to capture neurodynamic trends). We should, however, offer the caveat that the statistical basis of this result is unconventional. By utilizing permutation tests on pooled data, we compared against sampling distributions derived from the population of all possible trials from our particular set of participants, rather than the population of all possible participants. However, generalizations to an even less representative population (e.g., all neurons of a given type within a single monkey) are commonplace in neuroscience. Furthermore, there are several additional observations that support our conclusion that the forced-excursion model variant was best. In both model and data, the stimulus-locked profiles displayed a slope difference between easy and hard trials and no difference between speed and accuracy trials. Importantly, in the response-locked model predictions, the terminal amplitude differences between SAT conditions were reduced compared to the predictions retaining a free excursion, better resembling the neural signals. These findings support the hypothesis that differences induced by SAT instructions are explained by a global modulation of activity rather than by varying a single specific parameter/process.

Previous attempts to explain the SAT in the absence of variation in the decision boundary have done so by incorporating an urgency signal, i.e., an evidence-independent signal, which over time pushes the accumulation process toward a boundary (Cisek et al., 2009; Thura et al., 2012; Hawkins et al., 2015a,b). This integration of urgency is not dissimilar to our suggestion of an amplified accumulation process. Both approaches avoid a variation in response boundary by boosting accumulation in hasty decisions and make broadly analogous predictions regarding the SAT’s impact on accumulation profiles.

However, urgency models do differ mathematically from our forced-excursion model. While the former assume the addition of an independent and growing signal, i.e., a time-varying process, the latter is obtained by an adjustment of parameters derived from the more established free-excursion model, implying a time-invariant intrinsic amplification of the accumulation process induced by global changes of the system. To expand on this distinction (with the important caveat that urgency has been implemented in different ways by different authors), urgency may be implemented as the addition of an evidence-independent signal at each time step, with this signal growing over time (Hanks et al., 2014), or as the multiplication of evidence by such a signal (Ditterich, 2006), in which case accumulation noise is also subject to this time-varying gain. In the latter approach, the integration of evidence over time may additionally be deliberately downplayed via (very) leaky integration (Cisek et al., 2009). By contrast, our modeling instead captured the SAT by amplifying both signal and noise in a constant manner throughout the decision (with noise even amplified before the onset of the imperative stimulus, via the *S_z_*parameter). This is what we mean here by neural gain modulation: the amplification of both signal and noise in a time-independent manner. Note that the way starting-point noise was implemented here implies that it effectively conflates mean starting point with start-point variability (see methods/results). In this sense, our “fixed-excursion” terminology is a slight misnomer, as some part of our model’s ability to explain the SAT in both behavioral and neural data is still dependent on a reduction in excursion, but several other parameters also play a role, and the decision bound is fixed.

We wish to note that we are in no sense hostile to the concept of urgency. In fact, we tested urgency models as an additional exploratory analysis, but opted not to include these results for reasons of brevity and clarity. We implemented two kinds of urgency model, with a linear urgency signal proving more successful. This model was about as good as those we present here when fitting our behavioral data (it provided a better fit in the EEG experiment, but a worse one in the TMS experiment). For neurodynamic data, it performed very similarly to our forced-excursion model in the EEG experiment. Its ability to capture these data in the TMS experiment lay approximately mid-way between our forced and free-excursion classic models but did not differ significantly from either one. Indeed, we find the concept of “urgency” to be a useful one that somewhat overlaps our “neural gain” hypothesis and finds support in the neuroscientific literature (Thura and Cisek, 2017). Therefore, we do not claim that our model is better supported than urgency models, either here or in general. However, since a number of studies evaluating the concept of an urgency signal have been unable to support it, suggesting instead that standard sequential sampling models can fully account for all behavioral data (Balci et al., 2011; Karşılar et al., 2014; Hawkins et al., 2015a,b), we propose that forced-excursion model variants should at least be considered as an appropriate alternative to urgency signals, reconciling decades of model-based support for decision boundary variation with recent neural evidence.

Although we have argued that the simulated accumulation profiles of the forced-excursion model closely resemble both of our neural signals, supporting the notion of a global modulation of activity as the underlying mechanism explaining the SAT, there are nonetheless some differences between the empirical and simulated profiles. However, any model is a simplified approximation of the true neurocognitive mechanisms and is unlikely to perfectly simulate any given process. This is particularly the case for neural signals which inherently have a low signal-to-noise ratio, such as ERPs and in particular the MEP signal. Somewhat limited signal quality is however typical for experiments of this nature (O’Connell et al., 2012; Hadar et al., 2016), and we used large numbers of trials in both experiments, producing demonstrably interpretable neural signals. We would argue that the correspondence between model predictions and neural data, both here and elsewhere, is remarkable, given a class of models originally conceived to have a largely behavioral scope (Luce, 1986).

All neuroscientific methods have limitations. For example, our MEP signal is derived from a technique that both records and perturbs neural activity, with implications that are difficult to precisely predict (Hadar et al., 2016). However, methodological triangulation is an established approach to building a convincing body of evidence. Here, we obtained converging evidence from two fundamentally different signals, as both corticospinal excitability and a parietal ERP displayed qualitatively similar findings. While there were small practical differences between the experiments (e.g., one vs multiple sessions, bilateral vs unilateral responses), these are unlikely to qualitatively alter the accumulation process, and we have matched the simulation of model predictions to the processing of each neural signal to further reduce the impact of methodological differences on our interpretation. Although the suggestion that these signals represent decision accumulation is recent, both signals were modulated by the difficulty manipulation, supporting this account. Furthermore, previous research using more established neural correlates of decision-making in nonhuman primates has shown similar findings, suggesting widespread changes in activity when the SAT is manipulated (Heitz and Schall, 2012, 2013; Hanks et al., 2014). Collectively, we believe these neural findings warrant adjusting even a well-established model (by rescaling its parameters) given that the adjustment is purely conceptual and does not affect the behavioral fit.

A final potential concern relates to our decision to fit models to pooled data, i.e., at the group, rather than individual, level. Such collation may give rise to distorted RT distributions relative to the shape of underlying individual distributions. However, where comparisons have been made between the mean of sequential sampling model parameters derived from individual fits, and the same parameters derived from a single group fit, they have tended to suggest that the group fitting approach is not particularly problematic (Ratcliff et al., 2003, 2004). The procedure has been used in several recent papers (Dmochowski and Norcia, 2015; Twomey et al., 2015).

In conclusion, we set out to explore the neural mechanisms of the SAT by examining two neural correlates of the decision variable, an MEP signal reflecting corticospinal excitability and a parietal ERP component known as the CPP. The SAT is typically explained in sequential sampling models as a variation of the decision boundary. Here, we tested whether this variation is visible in neural activity or if it might instead be implemented through a mathematically equivalent gain change in neural activity. Our decision-related neural activity, independently sourced from two brain networks, resembled the accumulation profiles predicted by a forced-excursion model variant in which the boundary differences are transferred onto other decision parameters. Consistent with previous studies, our results therefore indicate that the SAT is implemented by global changes of neural activity, but that this conceptually important outcome does not necessarily invalidate traditional modeling approaches.

## Synthesis

Reviewing Editor: Satu Palva, University of Helsinki Decisions are customarily a result of the Reviewing Editor and the peer reviewers coming together and discussing their recommendations until a consensus is reached. When revisions are invited, a fact-based synthesis statement explaining their decision and outlining what is needed to prepare a revision will be listed below. Note: If this manuscript was transferred from JNeurosci and a decision was made to accept the manuscript without peer review, a brief statement to this effect will instead be what is listed below.

I have looked through your rebuttal letter to answer the comments raised by the reviewers in the Journal of Neuroscience submission and feel that you have satisfactory addressed all concerns raised.
